# Prevalence and Risk Factors Associated With Cesarean Section in Syria: A Cross‐Sectional Study of the Two Largest Health Centers

**DOI:** 10.1002/hsr2.70604

**Published:** 2025-04-01

**Authors:** Mohammed Abdulrazzak, Mohammed Moutaz Alshaghel, Moustafa Alhashemi, Wafik Mayo, Sana Oubari, Bakri Roumi Jamal, Muhammad Shahem Shammaa, Zahraa Jabas, Osama Al Horani, Mohamad Ali Keblawi, Hamdi Nawfal, Data Collection Group, Abdulkader Hajjar Muaffak, Dania Khudro, Joudy Karh Damour, Ahmad Jamil Kharrat, Louna Almesto Alabdullah, Ahmad Haj Asaad, Joud Khalili, Saleh Muzaiek, Kamar Antakli, Mohamed Aljamal, Sounar Shehada, Zacharia Sallah, Farah Altaher, Joudi Anadani, Hiba Marstawi, Sawsan Habash Ghattas, Esraa Jabas, Elham Otaky, Ahmad Alfardous Alazem, Majd Hanna, Abd Al Razak Kutneh, Salma Shebli, Sandi Aljoudeh, Batoul Daher, Khalil Alnumairi, Almuhannad Alawwad, Osama Akmik, Ihssan Hendeh, Ahmad Hassoun, Ghina Al‐Mustafa, Malaz Shammout, Mohammad Akram Abu Alshamat, Masa Bytamoni, Ahmad Othman, Nour Zuhair Abd Alaal, Abdullah Al Sharif, Mhd Aghiad Noureddin, Ahmad Al‐Farouh, Ahmad Al‐Esh, Mohamad Namor, Nafiza Martini

**Affiliations:** ^1^ Faculty of Medicine, University of Aleppo Aleppo Syria; ^2^ Faculty of Medicine, University of Damascus Damascus Syria; ^3^ Stemosis Association for Scientific Research Damascus Syria; ^4^ Pediatric Department Aleppo University Hospital Aleppo Syria; ^5^ Department of Obstetrics and Gynecology Aleppo University Hospital Aleppo Syria; ^6^ Faculty of Medicine, Syrian Private University Damascus Syria

**Keywords:** cesarian section, delivery, pregnancy, primary C‐Section, Syria

## Abstract

**Background and Aims:**

A cesarean section (CS) is a surgical procedure used during pregnancy and childbirth to ensure maternal and fetal well‐being. Global CS rates are increasing, with different studies demonstrating this trend. The purpose of this study, is to look into the prevalence of CS and its contributing factors in Syrian hospitals.

**Methods:**

A retrospective cross‐sectional study was conducted at Aleppo University Hospital and Damascus University Hospital in Syria. The data were collected from patients' medical records during the period between January and December 2021. The study population included women who gave birth at these hospitals in 2021. The study used a questionnaire with four domains: sociodemographic features, mother's history, birth history, newborn information, and delivery type with indications and complications. CS indications were evaluated using protocols from the Association of Scientific Medical Societies in Germany (AWMF). Statistical analysis was conducted using SPSS Statistics 25.0.

**Results:**

Among the deliveries, 47.4% were C‐sections, with slightly higher rate at Damascus. Population characteristics revealed differences in age, residency, smoking history, birth details, and associated medical conditions. The majority of participants were aged above 25 years old, rural residents, and nonsmokers. The primary CS cases were mainly medically indicated. Most C‐sections were repeat procedures (68%), with fetal distress being the most common indication. Aleppo had higher repeat C‐section rates (71.5% vs. 65.5% in Damascus). Most primary C‐sections were medically indicated (85.2%), while 14.8% were non‐indicated, often due to maternal requests or previous complicated births.

**Conclusion:**

This study sheds light on CS prevalence, indications, and influencing factors in Syria, contributing to the broader discourse on optimizing CS rates and improving maternal and neonatal outcomes. Further research is necessary to explore additional factors and interventions to curb unnecessary CS procedures.

**Abbreviations**
ACOG: American College of Obstetricians and Gynecologists
AUH: Aleppo University hospital
AWMF: Association of Scientific Medical Societies in Germany
BMI: body mass index
CDC: Centers for Disease Control and Prevention
CPD: cricopharyngeal dysfunction
CS: cesarean section
C‐sections: cesarean section
DUH: Damascus University Hospital
HELLP: haemolysis, elevated liver enzymes, and low platelets
NSTs: non‐stress tests
SPSS: Statistical Package for the Social Sciences
USA: United States of America
VBAC: vaginal birth after cesarean

## 1 Background

Cesarean section (CS) is a surgical procedure performed during pregnancy and childbirth as a method of maternal care, that involves making incisions in the mother's abdomen and uterus to deliver the fetus. It is utilized when vaginal delivery poses risks to the mother or baby, such as complications during labor or certain medical conditions [1].

Despite the ambiguity of CS indications, dystocia is responsible for one‐third of the CS rate. A previous cesarean section is also a significant factor, triggering nearly one‐third of all cases. Other major indications include fetal distress, breech presentation, premature fetus, antepartum hemorrhage, certain twin deliveries, maternal diabetes, ovarian and cervical malignancy, pre‐eclampsia, idiopathic thrombocytopenic purpura, and obstetric cholestasis [2]. Nevertheless, CS is increasing globally and now accounts for 18.6% of deliveries. Regardless of the medical factors, the rates of non‐indicated CS are rising due to financial, social, cultural and psychological factors [3, 4].

Although CS is a life‐saving procedure, it has numerous short‐term and long‐term health consequences for both mothers and their children. Mothers may experience complications such as uterine rupture, abnormal placentation, ectopic pregnancy, stillbirth, and preterm birth. Children born via CS could face higher risks of allergy, atopy, asthma, changes in immune development, and a decrease in the diversity of the intestinal gut microbiome [5].

Studies conducted in neighboring countries have indicated an increase in the number of cesarean sections performed. For example, a study in Lebanon [6] and another study in Tehran's teaching hospitals [7] both showed an increase in CS rates. The latest data available for Syria shows an increase from 12% in 1993 to 15% in 2002 [8]. For this reason, the current state of affairs in Syria is opaque and requires further investigation. The purpose of this study is to ascertain the prevalence of cesarean sections performed in Syrian hospitals as well as any potential risk factors.

## 2 Methods

### 2.1 Place of Study

The study was carried out in Obstetrics and Gynecology Department at the largest hospitals of the main Syrian cities: Damascus University Hospital (DUH), in the south, and Aleppo University hospital (AUH) in the north. These two hospitals located at the center of gouvernantes, and provides free general and specialized medical services for all patients from rural and urban areas.

### 2.2 Study Participants

The study population comprised women who had given birth in the mentioned hospitals during 2021. We included all deliveries that occurred in both hospitals during 2021. Women who were admitted for miscarriages with a gestational period of less than 24 weeks were excluded from the sample. Deliveries with important missing data were also excluded.

### 2.3 Study Design and Samples

This study is a hospital‐based retrospective cross‐sectional study. A group of trained collaborators obtained the study sample from patients' paper medical records at the mentioned hospitals, we have included all the samples during the period between January and December 2021. The sample size consisted of 4731 and 5064 participants in Aleppo and Damascus respectively, resulting in a total sample size of 9795.

### 2.4 Data Collection

The data were collected using a questionnaire composed of four domains. The first domain pertained to the sociodemographic features and the mother's history, including age, place of residence, blood type, and smoking habits, in addition to the medical, surgical, and gynecological history. Moreover, the second focused on the detailed birth history of the mother, encompassing the number of children, deliveries, miscarriages, pregnancies, and previous cesarean sections. The third domain focused on the newborn and their information, such as the number, sex, weight, gestational age, and Apgar score at the first and fifth minutes after birth. The last domain was dedicated to the type of delivery (vaginal or cesarean), indications, and any complications experienced by the baby or the mother.

We evaluated the indications of these cases based on the cesarean section indications defined by the guidelines of the Association of Scientific Medical Societies in Germany (AWMF). Absolute indications included chorioamnionitis, absolute disproportion, maternal pelvic deformity, fetal asphyxia, placenta previa, umbilical cord prolapse, uterine rupture, abnormal lie and presentation, as well as eclampsia and HELLP syndrome. Nevertheless, relative indications comprised pathological cardiotocography, previous cesarean section, and failure to progress in labor [9]. Any record with missing data except for pregnancy age, Apgar score and fetus weight was excluded from analysis.

### 2.5 Ethical Considerations

This study adhered to ethical guidelines, receiving approval from the Research Ethics Committees at Damascus and Aleppo Universities. Data were obtained from anonymized medical records, ensuring patient confidentiality. Informed consent was not required due to the retrospective nature of the study. However, to ensure patient confidentiality and data protection, all data were anonymized before analysis, and access was restricted to authorized personnel only.

### 2.6 Statistical Analysis

Data collection team have filled the questionnaires to Google forms to organize the process and create a secure resource. Google forms were exported to an excel file to clean and prepare the data for statistical analysis using SPSS 25.0 (SPSS Inc., Chicago, IL, USA). The results of the study are presented as frequencies and percentages. Apgar score and other continuous variables were modified to ordinal variables for clinical purposes. The chi‐square test was used to examine the relationship between governorate and other variables, as well as the differences between CS indications and governate. A significance level of alpha = 0.05 was used to determine statistical significance.

## 3 Results

**Figure 1:**
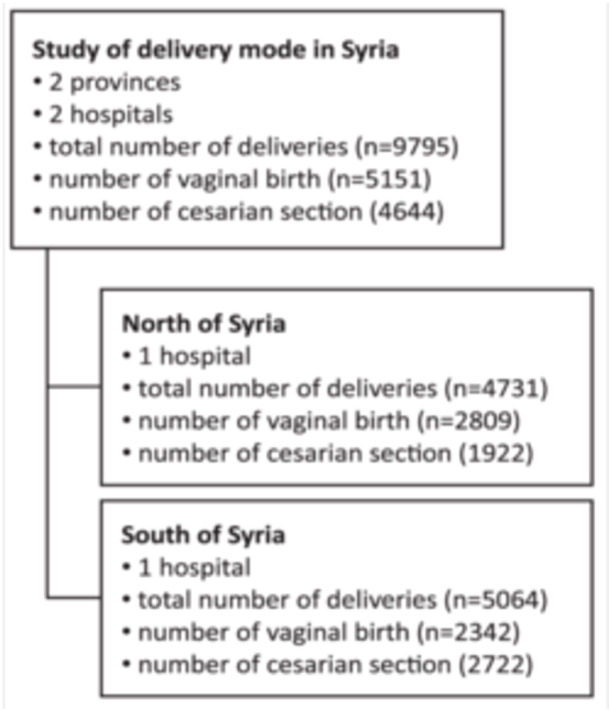
Study samples distribution.

A total of 9795 deliveries were recorded during the study period, with 4731 occurring in the northern region (Aleppo) and 5064 in the southern region (Damascus). In Aleppo, 2809 deliveries were vaginal, while 1922 were cesarean. Conversely, 2722 deliveries were in Damascus cesarean (Figure 1).

Women delivering in Damascus were significantly older than those delivering in Aleppo hospitals (*p* < 0.001). A higher proportion of women in Damascus originated from rural areas (85.5%) and were non‐smokers (92.2%), compared to women in Aleppo (*p* < 0.001). Additionally, 75% of women in Damascus had a Gravida of more than 1, with 27.4% having a Parity of 0. On the other hand, 78.1% of women in Aleppo had a Gravida of more than 1, with 25.7% having a Parity of 0. The history of abortion was significantly more in Aleppo (*p* < 0.001), while prior cesarean section was more common in Damascus (*p* < 0.001). Out of the sample population, 5.8% had heart diseases or hypertension, 1.1% had thyroid gland disease, and 11.4% had a history of surgery. There were no significant differences observed in terms of birth sex and plurality between the northern and southern regions. However, the rate of cesarean births was significantly higher in the south compared to the north (*p* < 0.001), where vaginal births were more common. More detailed demographics of all sample size are presented in the first Table 1.

**Table 1:** Characteristics of study populations.

Parameter	All (*n* = 9795)	Aleppo (*n* = 4731)	Damascus (*n* = 5064)	*p* value
	*n* (%)	*n* (%)	*n* (%)	
Age				**< 0.001**
< 18	620 (6.3%)	398 (8.4%)	222 (4.4%)	
18–24	3923 (40.1%)	1872 (39.6%)	2051 (40.5%)	
25–34	3716 (37.9%)	1728 (36.5%)	1988 (39.3%)	
≥ 35	1536 (15.7%)	733 (15.5%)	803 (15.9%)	
Residency				**< 0.001**
Rural	7292 (74.4%)	2959 (62.5%)	4333 (85.5%)	
Urban	2503 (25.5%)	1772 (37.5%)	731 (14.4%)	
Smoke				**< 0.001**
Non‐smokers	8863 (90.4%)	4193 (88.6%)	4670 (92.2%)	
Smokers	932 (9.5%)	538 (11.4%)	394 (7.7%)	
Gravida				**< 0.001**
1	2303 (23.5%)	1035 (21.9%)	1268 (25.0%)	
2	1660 (16.9%)	773 (16.3%)	887 (17.5%)	
3	1609 (16.4%)	701 (14.8%)	908 (17.9%)	
4	1261 (12.9%)	554 (11.7%)	707 (14.0%)	
≥ 5	2962 (30.2%)	1668 (35.3%)	1294 (25.6%)	
Parity				**< 0.001**
0	2603 (26.6%)	1214 (25.7%)	1389 (27.4%)	
1	1988 (20.3%)	882 (18.6%)	1106 (21.8%)	
2	1824 (18.6%)	790 (16.7%)	1034 (20.4%)	
3	1247 (12.7%)	595 (12.6%)	652 (12.9%)	
4	784 (8.0%)	397 (8.4%)	387 (7.6%)	
≥ 5	1349 (13.8%)	853 (18.0%)	496 (9.8%)	
Alive				**< 0.001**
0	2733 (27.9%)	1281 (27.1%)	1452 (28.7%)	
1	2094 (21.4%)	923 (19.5%)	1171 (23.1%)	
2	1827 (18.7%)	819 (17.3%)	1008 (19.9%)	
3	1229 (12.5%)	589 (12.4%)	640 (12.6%)	
4	734 (7.5%)	378 (8.0%)	356 (7.0%)	
≥ 5	1178 (12.0%)	741 (15.7%)	437 (8.6%)	
History of abortion				**< 0.001**
No	6833 (69.8%)	3141 (66.4%)	3692 (72.9%)	
Yes	2962 (30.2%)	1590 (33.6%)	1372 (27.1%)	
Prior cesarian section				**< 0.001**
No	6245 (63.8%)	3178 (67.2%)	3067 (60.6%)	
Yes	3550 (36.2%)	1553 (32.8%)	1997 (39.4%)	
Prior cesarian section (NUM)				**< 0.001**
0	6245 (63.8%)	3178 (67.2%)	3067 (60.6%)	
1	1528 (15.6%)	611 (12.9%)	917 (18.1%)	
2	1011 (10.3%)	398 (8.4%)	613 (12.1%)	
3	579 (5.9%)	288 (6.1%)	291 (5.7%)	
≥ 4	432 (4.4%)	256 (5.4%)	176 (3.4%)	
Heart disease/hypertension	573 (5.8%)	316(6.7%)	257(5.1%)	
Thyroid gland disease	112 (1.1%)	42 (0.9%)	70 (1.3%)	
GDM/DM	61 (0.6%)	21 (0.4%)	40 (0.8%)	
Surgical history	1112 (11.4%)	362 (7.7%)	750 (14.8%)	
Birth sex				0.513
Male	4974 (50.1%)	2397 (50.4%)	2577 (50.4%)	
Female	4905 (49.9%)	2372 (49.6%)	2533 (49.6%)	
Plurality				0.374
Singleton	9567 (97.7%)	4615 (97.5%)	4952 (97.8%)	
Multiple	228 (2.3%)	116(2.5%)	112 (2.2%)	
Pregnancy age (missing: 66)				**< 0.001**
< 37	1937 (19.9%)	1363 (29.1%)	574 (11.4%)	
37–42	7791 (80.0%)	3324 (70.9%)	4467 (88.5%)	
42+	5 (0.1%)	0	5 (0.1%)	
Fetus weight (missing: 1604, Aleppo:1572/Damascus:32)				
< 2500	883 (10.8%)	307 (9.7%)	576 (11.4%)	
2500–4000	7124 (87.0%)	2753 (87.1%)	4371 (86.9%)	
> 4000	184 (2.2%)	99 (3.1%)	85 (1.7%)	
Apgar score in 5 min (Missing: 522, Aleppo:459/Damascus:63)				0.257
< 4	13 (0.1%)	5 (0.1%)	8 (0.2%)	
4–6	125 (1.3%)	49 (1.1%)	76 (1.5%)	
7–10	9135 (98.5%)	4218 (98.7%)	4917 (98.3%)	
Birth type				**< 0.001**
Vaginal	5151 (52.6%)	2809 (59.4%)	2342 (46.2%)	
Cesarean	4644 (47.4%)	1922 (40.6%)	2722 (53.8%)	

Out of a total of 4644 cesarean sections analyzed, 3157 (68%) were repeat cesarean sections, while 1487 (32.0%) were primary cesarean sections. In Aleppo, 1376 (71.5%) of the cesarean sections were repeat procedures, whereas 548 (28.5%) were primary cesarean sections. Conversely, in Damascus, 1783 (65.5%) of the cesarean sections were repeat procedures. The majority of primary cesarean sections (85.2%) had medical indications with a higher percentage of (88.1%) in Aleppo while (83.6%) in Damascus. On the other hand, the nonindicated CS were higher in Damascus (16.4%) in comparing with Aleppo (11.9%) with a total percentage (14.8%). The mother request was most common nonmedical‐indication in Damascus (5.6%) while in Aleppo precious baby (8.2%). However out of the medical indicated caesarian section was fetal distress with the highest common either in Aleppo (35.0%) or in Damascus (30.2%). Malpresentation were higher in Aleppo (34.5%) while in Damascus (24.9%). Never the less is CPD significantly higher in Damascus (10.2%) while in Aleppo (2.2%) (Table 2).

**Table 2:** The indications for cesarean section by regions.

Indication	All Cs (*n* = 4644)	Aleppo (*n* = 1922)	Damascus (*n* = 2722)	
	*n* (%)	*n* (%)	*n* (%)	
**Total cesarian section**	4644 (100%)	1922 (100%)	2722 (100%)	
Repeat cesarean section	3157 (68%)	1376 (71.5%)	1783 (65.5%)	
Primary cesarean section	1487 (32.0%)	548 (28.5%)	939 (34.5%)	

Indication for primary CS	**All (*n*** = **1487) *n* (%)**	**Aleppo (*n*** = **548) n (%)**	**Damascus (*n*** = **939) *n* (%)**	***p* value**
**Indicated cesarian section**	1267 (85.2%)	483 (88.1%)	784 (83.6)	
CPD	108 (7.3%)	12 (2.2%)	96 (10.2%)	< 0.001
Malpresentation	423 (28.4%)	189 (34.5%)	234 (24.9%)	0.001
Threat of uterine rupture	41 (2.8%)	12 (2.2%)	29 (3.1%)	0.314
Arrest and protraction disorders	163 (11.0%)	68 (12.4%)	95 (10.1%)	0.198
Placenta previa/abruptio/bleeding	90 (6.1%)	41 (7.5%)	49 (5.2%)	0.078
Fetal distress	476 (32.0%)	192 (35.0%)	284 (30.2%)	0.115
Pre‐eclampsia/eclampsia	62 (4.2%)	25 (4.6%)	37 (3.9%)	0.571
Cord prolapses	10 (0.7%)	3 (0.5%)	7 (0.7%)	0.653
Medical conditions	66 (4.4%)	32 (5.8%)	34 (3.6%)	5.05
Others	16 (1.1%)	7 (1.3%)	9 (1.0%)	0.567
**Nonindicated cesarian section**	220 (14.8%)	65 (11.9%)	155 (16.4%)	
Mother request	53 (3.6%)	0	53 (5.6%)	
Precious baby	94 (6.3%)	45 (8.2%)	49 (5.2%)	0.027
Mother age > 35	45 (3.0%)	13 (2.4%)	32 (3.4%)	0.268
Others	28 (1.9%)	7 (1.3%)	21 (2.2%)	0.194

Abbreviation: CPD, cricopharyngeal dysfunction.

**Figure 2:**
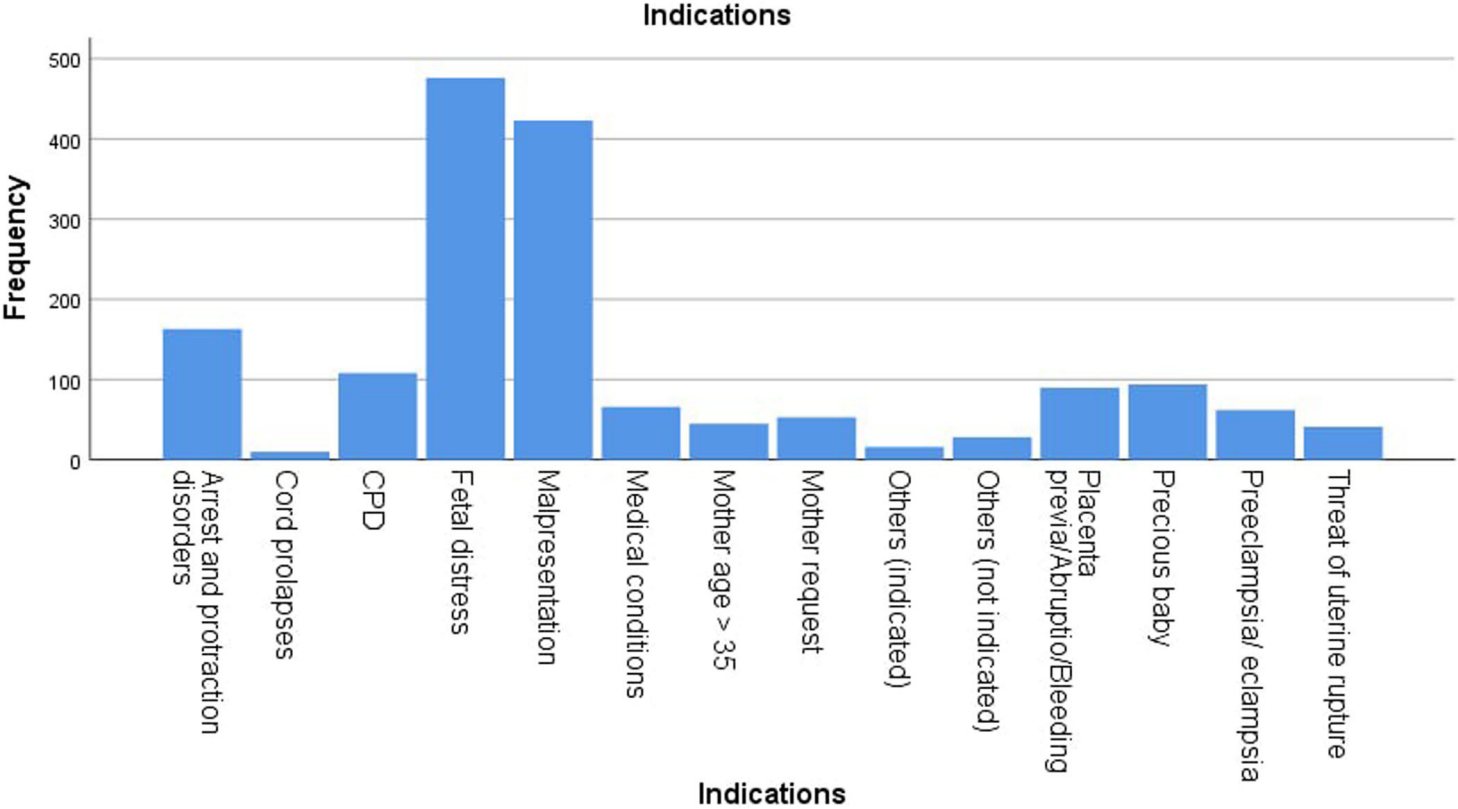
Primary cesarian sections indications among study population.

The second figure illuminates that the highest indications for cesarean sections were fetal distress and malpresentation with a big difference from the rest of the indications (Figure 2).

**Figure 3:**
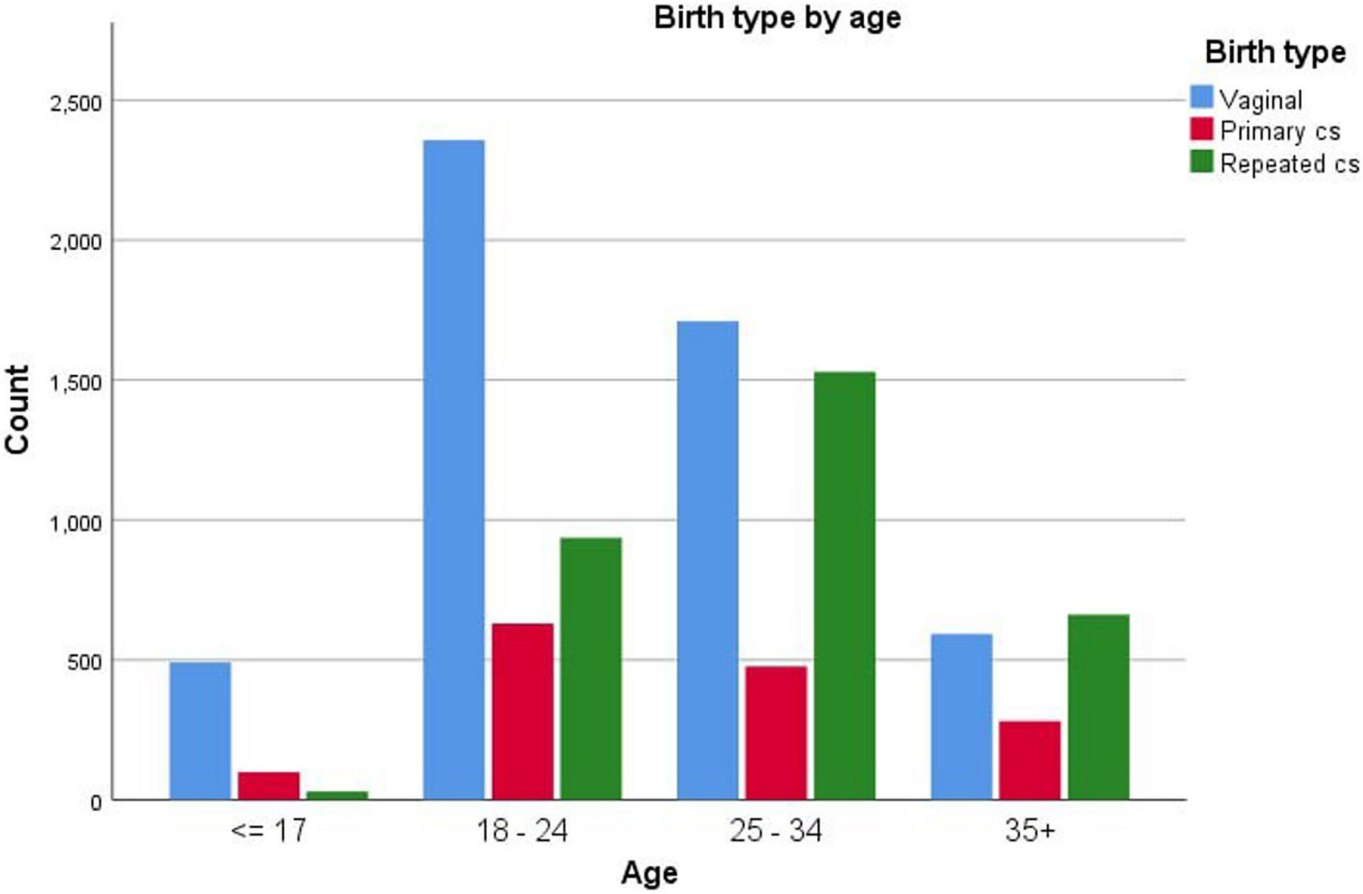
Mode of delivery among different age groups in study population.

It indicates that among women above 35, repeated cesarean sections were the most common mode of delivery, whereas vaginal delivery was the predominant mode in all other age groups. At the same time primary cesarean sections were the least common mode of delivery in women above 18, on the other hand repeated cesarean sections were with the lowest need in women under 18 (Figure 3).

Among the 3157 repeat cesarean sections, 1529 were performed on women between the ages of 25 and 34, while only 30 were performed on women < 18. For primary cesarean sections, 630 out of 1487 were conducted on women aged between 18 and 24, and only 99 were performed on women < 18. Out of the Indicated caesarian section, Fetal distress was the most common indication for cesarean section in women < 18 (41.4%) and between 18 and 24 (39.0%). However, malpresentation was the most observed indication in both women between 24 and 34 (31.6%) and +35 (23.1%). Moreover, pre‐eclampsia/eclampsia is significantly higher in women < 18 (9.1%) in comparing the other groups. Regarding nonindicated cesarean sections, women aged +35 were with the highest rate. The most common cause in all groups was precious baby (Table 3).

**Table 3:** The indications for cesarean section by age groups.

Parameter	All Cs (*n* = 4644)	< 18 (*n* = 129)	18–24 (*n* = 1566)	25–34 (*n* = 2006)	+35 (*n* = 943)
	*n* (%)	*n* (%)	*n* (%)	*n* (%)	*n* (%)
**Total cesarian section**	4644 (100%)	129 (100%)	1566 (100%)	2006 (100%)	943 (100%)
Repeat cesarean section	3157 (68.0%)	30 (23.3%)	936 (60.0%)	1529 (76.2%)	662 (70.2)
Primary cesarean section	1487 (32.0%)	99 (76.7%)	630 (40.0%)	477 (23.8%)	281 (29.8)

Indication for primary CS	All (*n* = 1487)	< 18 (*n* = 99)	18–24 (*n* = 630)	25–34 (*n* = 477)	+35 (*n* = 281)
	*n* (%)	*n* (%)	*n* (%)	*n* (%)	n (%)
**Indicated cesarian section**	1267 (85.2%)	95 (96.0%)	567 (90.1%)	429 (90.0%)	176 (62.7%)
CPD	108 (7.3%)	7 (7.14%)	62 (9.8%)	30 (6.3%)	9 (3.2%)
Malpresentation	423 (28.4%)	31 (31.3%)	176 (27.9%)	151 (31.6%)	65 (23.1%)
Threat of uterine rupture	41 (2.8%)	2 (2.0%)	6 (0.9%)	17 (3.6%)	16 (5.7%)
Arrest and protraction disorders	163 (11.0%)	8 (8.1%)	72 (11.4%)	54 (11.3%)	29 (10.3%)
Placenta previa/abruptio/bleeding	90 (6.1%)	2 (2.0%)	31 (4.9%)	39 (8.2%)	18 (6.4%)
Fetal distress	476 (32.0%)	41 (41.4%)	246 (39.0%)	134 (28.1%)	55 (19.6%)
Pre‐eclampsia/eclampsia	62 (4.2%)	9 (9.1%)	17 (2.7%)	17 (3.6%)	19 (6.8%)
Cord prolapses	10 (0.7%)	1 (1.0%)	2 (0.3%)	4 (0.8%)	3 (1.0%)
Medical conditions	66 (4.4%)	2 (2.0%)	25 (3.9%)	22 (4.6%)	17 (6.1%)
Others	16 (1.1%)	1 (1.0%)	4 (0.6%)	9 (1.9%)	2 (0.7%)
**Nonindicated cesarian section**	220 (14.8%)	4 (4.0%)	63 (9.9%)	48 (10.0%)	105 (37.3%)
Mother request	53 (3.6%)	2 (2.0%)	25 (3.9%)	17 (3.5%)	9 (3.2%)
Precious baby	94 (6.3%)	2 (2.0%)	37 (5.9%)	28 (5.9%)	27 (9.6%)
Mother age > 35	45 (3.0%)	0	0	0	45 (16.0%)
Others	28 (1.9%)	0	1 (0.15%)	3 (0.6%)	24 (8.5%)

## 4 Discussion

The C‐section rate has significantly increased worldwide in the last three decades, as it enhances pregnancy outcomes, and decreases mortality and morbidity among both mothers and babies. However, overuse has limited these benefits [10]. Moreover, the evidence has not shown any benefits of C‐sections for women and babies without any medical indication. Over the past few years, experts in the medical field and governments have shown concern regarding the increase in the frequency of C‐section deliveries and the possible adverse effects on the health of both the mother and the baby [4].

The presented paper is the largest to demonstrate the C‐section phenomenon and determine its dimensions in Syria. The aim of this study is to estimate the prevalence of C‐sections, and their medical and nonmedical indications, and determine their related factors in the Syrian community. A high C‐section rate is a serious public health issue, and determining its indication is the first step in designing programs to control these rates.

Our main findings show a high rate of C‐section procedures among the Syrian community 47.4%, which is close to the C‐section rate in Lebanon 48%, and less than the rates in Egypt and Saudi Arabia 53% and 55%, respectively [6, 11, 12]. However, the findings in other parts of the world were familiar to those in Arab countries, Xin et al. reported in the largest delivery survey in China that 54.5% of all deliveries were C‐sections, while it was 32.8% of all deliveries in the United States according to the CDC National Vital Statistics report [13, 14]. The global healthcare community has recommended a desirable range for the frequency of cesarean sections, which is typically between 10% and 15% [4, 15].

The impact of nonclinical factors, such as mother's socio‐demographic characteristics on the rates of C‐sections is significant [16]. Several studies have studied the association between maternal age, economic status, and educational level. Sociodemographic factors were not fully documented in our data, such as educational level and economic status. Hence, the primary focus was on the “maternal age” factor, which revealed an increased risk of cesarean birth with increasing maternal age. Women under 35 years of age had the highest number of deliveries of both types, while pregnant women over 35 years of age showed an elevated risk for C‐sections [12].

The main justification for the performance of C‐section is the optimal outcome for the survival and well‐being of both the mother and newborn. The indications for the procedure can be broadly classified as absolute and relative indications [9]. While C‐sections are primarily performed for medical reasons, there are cases where a C‐section may be requested for nonmedical reasons. Some women may prefer a C‐section for personal reasons, such as a fear of vaginal delivery or a desire for greater control over the timing of the delivery. However, this is generally discouraged as C‐sections carry risks and are major surgeries [17]. The American College of Obstetricians and Gynecologists (ACOG) recommends that the decision to perform cesarean delivery should be based on the safest mode of delivery for the woman and her fetus [18].

In this study, the reason for C‐section 68% was a previous cesarean section, and this indication was the highest in Egypt with 50% of all C‐section cases. However, it was cited in Germany in less than a quarter of all C‐sections [9, 11]. Moreover, 36.2% of the study sample had at least one previous C‐section, and 20.6% had more than one. The inclination towards repeat cesarean deliveries seems to stem from the feeling of safety among physicians and mothers. When a woman has had a previous C‐section, there are two options for delivering subsequent babies: either a Vaginal Birth After Cesarean (VBAC), or a repeat C‐section [19]. While VBAC could be a safe and viable option for many women, some may opt for a repeat C‐section for various reasons, such as a high‐risk pregnancy or a previous difficult delivery. However, repeated C‐sections could create some concerns as the potential complications during surgery. There is an increased risk of uterine rupture, which can be life‐threatening for both the mother and baby. This risk increases with each subsequent C‐section [20].

On the other hand, 32% of all C‐sections were primary cesarean occurred for the first time. A total of 85.2% was indicated for medical reasons. However, the most common reason for medically indicated cesarean was fetal distress 32.0% and malpresentation 28.4%. Among different age groups, fetal distress was the highest indication in the first and second age groups, whereas malpresentation was the highest indication in the third and fourth age groups.

Fetal distress was the most commonly diagnosed indication in China; however, it was significantly lower than that in our study, and higher than that in the United States. The same study suggests that the rate for this indication was much higher in tertiary care hospitals than in secondary ones [14]. The possible explanation for these findings is the excessive utilization of technology in Syria for low‐risk pregnancies. In several medical facilities, it is customary to administer weekly non‐stress tests (NSTs) to all expectant mothers in the third trimester, even if they are healthy. However, these tests have a limited positive predictive value when employed in populations with low risk, which may lead to unnecessary cesarean sections being performed under the pretext of “fetal distress” [21]. Huang et al. [22] in their study that recruited 2326 women showed a significant association between the number of antenatal ultrasound scans and c‐section rates in rural areas of China.

In this study, only 14.8% of all C‐sections were non‐indicated. The first indication was the mother's request for a precious baby at 6.3%, followed by the mother's request with no specific reason at 3.6%. This is the result of the strict rules obligated by the hospitals to control cesarian section rate. Moreover, due to the lack of complete clinical data on the cases, a number of C‐section that were classified as “indicated” may actually be “non‐indicated.” Moreover, there are some diagnoses that are currently classified as “indicated” and not universally accepted as evidence‐based indications for cesarean section, such as multiple gestation, pre‐eclampsia/eclampsia/HELLP syndrome, third‐trimester bleeding, and suspected macrosomia. This suggests that there might be some misclassification bias and as a result, the count of nonindicated C‐sections may have been underestimated [14]. Cesarean section on maternal request was the most commonly recorded reason for C‐section in many parts of the world [23].

The increasing prevalence of C‐sections worldwide has led to a growing debate about the knowledge and attitudes surrounding the procedure. One of the key factors that influence attitudes toward C‐sections is knowledge about the risks and benefits of the procedure. Studies have shown that women who are well‐informed about C‐sections are more likely to have a positive attitude toward the procedure, while those with limited knowledge may have more negative perceptions [24, 25]. some women may believe that C‐sections are safer than vaginal deliveries, but in reality, C‐sections are associated with a higher risk of certain complications such as infections, bleeding, and blood clots [26]. On the other hand, women who are aware of the potential benefits of C‐sections, such as avoiding other complications during labor, may be more open to the procedure [5]. In addition, attitudes toward C‐sections can also be affected by cultural and social factors. In some cultures, C‐sections are stigmatized as a “lazy” or “unnatural” way of giving birth, which can lead to negative attitudes and even discrimination against women who have had the procedure. Similarly, social pressure to have a “natural” birth or to conform to certain expectations about motherhood can also influence attitudes toward C‐sections [27].

Healthcare providers can also play a role in shaping women's perceptions of C‐sections. Some may have a biased attitude toward C‐sections, which can impact the information they give to patients and the recommendations they provide [28]. In accordance with established guidelines, it is recommended to discuss the mode of birth with all pregnant women at an early stage. These discussions should cover different aspects, as approximately 25%–30% of women may undergo a cesarean birth and various factors that increase the likelihood of cesarean birth, such as advanced maternal age and an elevated BMI. Furthermore, it is crucial to discuss the details of the cesarean birth procedure and its implications for future pregnancies and subsequent births, whether vaginal or cesarean [29].

There are many examples of governmental procedures aimed at controlling C‐section rates across the world. The US Department of Health and Human Services launched the “Healthy People 2020” initiative, which included a goal to reduce the rate of C‐sections among low‐risk women [30]. The initiative included strategies for healthcare providers and hospitals to promote vaginal birth and reduce the rate of unnecessary cesarean sections. In 2000, the Brazilian Ministry of Health launched the “Childbirth and Humanization” program, which included educational campaigns for women and healthcare providers, as well as financial incentives for hospitals that reduced their cesarean rates [31].

One potential way to reduce the number of unnecessary C‐sections performed is by utilizing globally recognized criteria that are based on empirical evidence to establish clinical guidelines for obstetric management. Additionally, reducing cesarean section rates may be achieved by putting rules aimed at addressing medical malpractice and prioritizing the experience of vaginal birth.

Our findings highlight the pressing need to address the high rates of cesarean sections in Syria and similar settings. While the rate of 47.4% in our study aligns with regional trends, it far exceeds the WHO‐recommended optimal range of 10%–15%, underscoring a significant public health concern. The predominance of repeat C‐sections (68%) and the substantial proportion of primary C‐sections (32%) emphasize the necessity of strengthening clinical decision‐making processes. Implementing evidence‐based guidelines, such as those proposed by ACOG, could help ensure that C‐sections are performed only when medically indicated.

Moreover, the high prevalence of fetal distress and malpresentation as leading indications for primary C‐sections in our study points to the potential overuse of technology, such as nonstress tests, in low‐risk pregnancies. Future research should investigate the predictive value of these diagnostic tools and their role in unnecessary surgical interventions. Additionally, educational campaigns targeting both healthcare providers and expectant mothers are critical to raising awareness of the risks and benefits of different delivery modes, encouraging informed decision‐making.

### 4.1 Study Limitation

Finally, it is crucial to note the limitations of our study, as the largest limitation was that the centers recruited in the study were not randomly selected, which could lead to selection bias. Moreover, our study includes only public hospitals, which provide free medical services. This may lead to polarizing the high‐risk pregnancies for cesarean section. Additionally, these centers are committed to making cesarean sections just for medical indications, with a little exception. This will restrict the ability to generalize our results to smaller centers. We obtained our data manually from the patient's files because there is no electronic record or database to include it.

## 5 Conclusion

This study explores factors affecting cesarean section rates, providing valuable insights for healthcare policymakers and providers. It emphasizes the need to regulate rates, promote evidence‐based practices, and improve maternal and infant outcomes. Further research is needed to explore other interventions. At a systemic level, integrating electronic medical records could enhance data accuracy and facilitate ongoing surveillance of obstetric outcomes. Finally, further research is needed to explore nonclinical factors, such as cultural attitudes and healthcare provider biases, which may contribute to the high C‐section rates, as well as the impact of policy interventions in diverse healthcare settings.

## Author Contributions

**Mohammed Abdulrazzak:** conceptualization, investigation, writing – original draft, methodology, validation, writing – review and editing, project administration, data curation, supervision, resources, visualization. **Mohammed Moutaz Alshaghel:** writing–original draft, writing – review and editing, project administration, data curation, resources, methodology, visualization, formal analysis, software. **Moustafa Alhashemi:** writing – original draft, writing – review and editing, methodology, software, data curation, project administration, resources, supervision. **Wafik Mayo:** investigation, writing – original draft, writing – review and editing, project administration, data curation, resources, methodology. **Sana Oubari:** writing – original draft, writing – review and editing, data curation, resources, methodology. **Bakri Roumi Jamal:** writing – original draft, writing – review and editing, data curation, resources. **Muhammad Shahem Shammaa:** writing – original draft, writing – review and editing, data curation, resources, methodology. **Zahraa Jabas:** methodology, writing – review and editing, writing – original draft, data curation, resources. **Osama Al Horani:** writing – original draft, writing – review and editing, data curation, resources. **Mohamad Ali Keblawi:** supervision, writing–original draft, writing – review and editing, resources. **Hamdi Nawfal:** supervision, writing – review and editing.

## Ethics Statement

The study protocol was approved by the Research Ethics Committee at Damascus, and Aleppo Universities, and the ethical committees in the concerned hospitals.

## Consent

Informed consent was not required due to the retrospective nature of the study. However, to ensure patient confidentiality and data protection, all data were anonymized before analysis, and access was restricted to authorized personnel only.

## Conflicts of Interest

The authors declare no conflicts of interest.

## Transparency Statement

The lead author Mohammed Abdulrazzak affirms that this manuscript is an honest, accurate, and transparent account of the study being reported; that no important aspects of the study have been omitted; and that any discrepancies from the study as planned (and, if relevant, registered) have been explained.

## Data Availability

The data that support the findings of this study are available from the corresponding author upon reasonable request.
